# The illusion of immediacy: on the need for human synchronization in data-intensive medicine

**DOI:** 10.3389/fsoc.2023.1120946

**Published:** 2023-08-03

**Authors:** Martina von Arx

**Affiliations:** Section of Biology, University of Geneva, Geneva, Switzerland

**Keywords:** personalized medicine, data-intensive medicine, patient remote monitoring, telecare, temporalities, synchronization, immediacy, Switzerland

## Abstract

Medical practice is increasingly shaped by big data sets and less by patient narratives. Data-intensive medicine promises to directly connect the patients with the clinic. Instead of medical examinations taking place at bedside and discrete moments, sensor-based technologies continuously monitor a certain body parameter and automatically transfer the data via a telemedical system. Based on a qualitative study of remote cardiac monitoring, I explore how the uncoupling of processes that used to happen in one place, changes the way diagnosis is made. Using ethnographic observations and semi-structured interviews with patients and tele-nurses of two university hospitals in Switzerland, I describe remote cardiac monitoring as a data network. The perception of being constantly connected to the hospital resulted in a reassuring effect among patients and healthcare professionals. Moreover, the notion of an automatically synchronized data network led patients to expect immediate feedback from the hospital as soon as an irregularity was detected. However, it obscured the fact that although the inserted sensor monitors the heart around the clock, the data is transmitted only once a day, and the tele-nurses only work during office hours, from Monday to Friday. I call this misperception “illusion of immediacy”. It takes time to accurately correlate and interpret a recorded episode with other types of data, such as the last hospital visit, comorbidities, and/or the actual situation in which the recording was made. Accordingly, tele-nurses and cardiologists play a central and privileged role in the data network. The findings highlight the importance of synchronizing the different temporalities that coexist in the patient remote monitoring data network in order to generate meaningful knowledge that ultimately leads to a diagnosis.

## Introduction

The heart beats about 60–100 times per minute in a healthy human. Blood rich in oxygen and nutrients circulates through the body with every heartbeat. The heart rhythm, or its palpable effect, the pulse, are easy to detect vital signs. However, the human body does not always work like a clock, and the heartbeat can get out of sync. Strong feelings or physical exercise might be the cause for short-term changes in the heart rhythm. Such experiences are captured by idioms like “losing one's heart to someone” or the heart “racing a mile a minute”. Most heart arrhythmias do not represent an immediate danger to life. Nevertheless, experiencing a heart out of sync or the associated symptoms, for example fainting, can be scary, especially if there is no obvious cause. Quite like earthquakes, heart arrhythmias can occur at indefinite intervals of varying duration, and sometimes even without noticeable symptoms (Jones, 2013). As a result, they are almost impossible to detect during regular office or hospital visits. This is where my story begins (Moers, 2008).

To detect such an elusive but potentially life-threatening condition, the heart must be monitored continuously. In countries like Switzerland, where telecare is covered by basic health insurance, cardiologists may recommend the insertion of a small, remotely connected device called a cardiac monitor. Instead of data snapshots of a given patient, at a given time and place, inherent in traditional calendar-based follow-up, long-term remote cardiac monitoring creates a continuous flow of data. Using several algorithms to analyze the electrical signals detected by the two sensors of the cardiac monitor, the continuous monitoring reports any event that deviates from the programmed thresholds. Thus, measurements that are within the norm will no longer appear in the patients' medical records, because the algorithms will only report what is outside the norm. This is a major difference from calendar-based measurements, which are based on a specific point in time (e.g., every 3 months) determined by evidence-based medical standards or the cardiologists' experience. Therefore, continuous monitoring delivers unprecedented big data sets for long-term intrapersonal data comparison (Sysling, 2020), thereby inducing a shift in how, when and where patient data are collected, analyzed and interpreted.

This kind of data-intensive medical practice has profound implications for how, when, and where a diagnosis is made. In a traditional medical appointment, the patient and his or her narrative, the cardiologist and his or her expertise, the device, and likely the recordings, are all in one place. This configuration allows the patient and cardiologist to immediately comment and discuss possible findings or agree on the next steps (e.g., call the patient as soon as the test results are available). In a remote monitoring system, the device follows the patient wherever he or she goes, the cardiologist works his or her usual shifts at the hospital, and the recordings are simultaneously with the patient, in the data cloud, and at the hospital. The processes of data collection, analysis, and interpretation that used to be part of the traditional doctor-patient appointment, become uncoupled.

Previous studies mostly focused on the obvious spatial uncoupling of healthcare induced by surveillance medicine (Armstrong, 1995) or telecare (Oudshoorn, 2011; Pols, 2012). However, in addition to the important questions related to the spatial distribution of the different actors, who were previously reunited in a medical site according to a calendar-based schedule, the remote monitoring of patients raises another problem that has not yet received much scientific attention: the temporal uncoupling.

Data-intensive technologies are often promoted with the promise to deliver timely diagnosis through continuous data monitoring thereby anticipating bad outcomes. From the cardiac monitor to biobanks or wearables—not only medical practice, but health in general is increasingly shaped by big data sets (Ruckenstein and Schull, 2017). These sets are getting bigger on the one hand because measurement tools are multiplying, starting in the mid-nineteenth century with acoustic, visual, chemical, and eventually sensor-based technologies aimed at ever more detailed and comprehensive measurements. On the other hand, they are getting bigger because measurement intervals are getting shorter or even disappearing altogether, as is the case with patient remote monitoring. The implicit promise is that the larger and more comprehensive the data sets, the better and more personalized the healthcare (Rosenberg, 2002). With the proliferation of such data-intensive biomedical technologies, the concepts of “personalized,” “stratified,” and “precision” medicine have emerged in the scientific and policy landscape over the past 20 years (Mackintosh and Armstrong, 2020; Cesario et al., 2021). The most commonly used term, “personalized medicine”, adopts the above vision and promises to use large integrated datasets to deliver the right treatment to the right patient at the right time (Petersen, 2018; Erikainen and Chan, 2019). However, contrary to what the term suggests, the main feature of “personalized medicine” is not the person, but the big data sets (Prainsack, 2017; Hoeyer, 2019). Today, the vision of “personalized medicine” is based on a *technoscientific holism* consisting of an integrative aggregate of all quantifiable units of human life (Vogt et al., 2016). However, in order to construct and connect all these different types of data, they must be easily transferable from one context to another. Just as mathematics has become the universal language in public and scientific discourse, datafication is the answer to a medical practice that increasingly resembles a data network (Porter, 1995). Such a network-like character is made possible by prioritizing digital, quantified, and computable evidence while downgrading unstructured data, narratives, and embodied experience (Prainsack, 2017; Hoeyer, 2019). Contrary to unstructured information, quantified evidence is easier to collect, process, and share remotely. Therefore, Theodore (1995) called quantification a technology of distance fostering global networks rather than local communities. Thinking of medical practice as a data network is not only about the fact that data travels easily. It's also about data being not just in one physical place, but in multiple places at once. Even if remote cardiac monitoring data are stored by the biomedical companies in the Netherlands, France, or Germany, it can be accessed from other places if access rights are granted (Maillard et al., 2014). In this way, digital data and the knowledge it contains are no longer tied to one place, but are a property of the network (Weinberger, 2011).

Already by the mid-twentieth century, biophysicist Norman “Jeff” Holter dreamt of a system continuously collecting, transmitting, and analyzing all types of physiological data to detect potentially hidden diseases in the seemingly normal measurement variations (Greene, 2022). Enthusiastic about emerging transmission technologies, he developed the first portable cardiac monitor in 1949 that could record an electrocardiogram “on the go” (Kalahasty et al., 2013). Initially worn as a bulky backpack, the device soon became smaller, and his dreams of continuous monitoring became more realistic. Today's “Holter” monitors consist of three leads attached to the skin of the chest and a recording box, usually attached to a necklace or belt, that monitors for 24 h, 48 h, or 7–14 days. Patients wear such a device for the desired period, then return the recorder box to the hospital, where the data are analyzed, and the results are reported to the patients. Other conventional tests include x-rays, echocardiography, or an electrophysiology study (Deftereos et al., 2016; Schweizerische Herzstiftung, 2022). If these examinations or short-term cardiac monitoring do not yield results, long-term remote cardiac monitoring offers a possibility of detecting the suspected arrhythmia.

Current cardiac monitors are barely the size of a triple-A battery, weigh about 4 grams, and are made of titanium, sapphire, parylene, silicone, or iridium components that protect the electronics and make them compatible with human tissue. In Switzerland, the insertion (and later removal) of the cardiac monitor and remote monitoring are covered by basic health insurance. Once inserted, it is not possible for patients to interrupt or stop the continuous monitoring. Usually, the cardiac monitor is connected to a telemedical system automatically transmitting the recorded data once a day. The battery life of the cardiac monitor lasts between 3 and 5 years. The cardiac monitor typically gets removed once a diagnosis is established or when the battery life ends.

The number of patients having cardiac monitor in Switzerland is not tracked separately to other cardiac implants. Most devices under telemedical monitoring serve therapeutic purposes, which is for example the case for defibrillators. Based on the interviews done for this study, the two studied hospitals monitor about 1,000 patients for diagnostic reasons.

Remote cardiac monitoring differs from conventional methods in that the measurements are no longer bound to a specific space and timeframe. Hence, examining the temporal uncoupling of data collection, analysis, and interpretation is at the core of this article. Different time frames and data types must be in sync to render them meaningful for cardiologists and patients. A condition which is no longer given when these processes do not happen in the same place as it was the case for traditional follow-ups. Hence, the aim of this article is to illustrate how patient remote monitoring is reconfiguring the way diagnosis is made, based on a qualitative study of remote cardiac monitoring conducted in Switzerland. To highlight the challenges of synchronizing what has become uncoupled by remote monitoring, the analysis will frame remote cardiac monitoring as a data network. Although the imaginary of a data network conveys the idea of constant synchronicity, I will show that data transmission, processing, and medical interpretation introduce time lags that lead to misunderstandings between patients and healthcare professionals.

## Materials and methods

The qualitative study was conducted at two university hospitals in different linguistic regions of Switzerland from October 2020 to July 2022. Ethnographic observations were conducted in the telemedical unit and during the ambulatory insertion procedures. The latter served also to recruit patients for interviews. Semi-structured interviews were conducted with patients (women = 12, men = 16), nurses specialized in remote cardiac monitoring (*n* = 7), cardiologists (*n* = 9), and industry representatives (*n* = 4). The median age of the patients was 62 years, with the youngest being 21 years and the oldest being 85 years. Their socioeconomic backgrounds varied from truck driver to secretary to director of a retirement home. Of the 28 patients interviewed, 15 were included in a longitudinal follow-up consisting of two interviews. The first interview took place 3–8 weeks after device insertion. A second interview was conducted 6–8 months after insertion. Patients were asked to provide an additional update on their experience by letter in the summer of 2022. Three patients dropped out after the first interview. The other 10 patients participated in a retrospective interview after the cardiac monitor was removed, either when a diagnosis was made or when the battery was exhausted. Partners of the patients were present in three interview situations. The researcher was unable to recruit a patient who had refused to have a cardiac monitor implanted. This article focuses on the data collected during the ethnographic observations and interviews with patients and nurses.

Data collection was affected by the COVID-19 pandemic, which significantly prolonged fieldwork. Correspondingly, the opportunities for ethnographic observations were limited by the regulated access to hospitals for non-medical staff. Although originally planned as face-to-face conversations, all of the first wave interviews were conducted remotely (by telephone or videoconference), with the exception of one person who insisted on being interviewed at her home. Once vaccination was available to all adults in Switzerland, it was up to the participants to decide whether they preferred a face-to-face interview or a remote form of communication. Most opted for a face-to-face interview.

Interviews were transcribed verbatim. Ethnographic notes were taken by hand and then transcribed on the computer. Data were coded using Atlas.ti software and applying reflexive thematic analysis (Braun and Clarke, 2021). Some codes were derived from the interview guide. Others were created while listening to the first wave of patient interviews. Several codes indicating the various forms of absence within the data network of remote cardiac monitoring (e.g., feedback, data access, cardiologists) were combined under the theme “illusion of immediacy” as perceived by patients. This theme was the starting point for this article.

The study was reviewed and approved by the “Commission cantonale d'éthique de la recherche sur l'être humain” (Cantonal commission on ethics in human research of the canton Vaud) in September 2020. Patients and healthcare professionals provided their written informed consent to participate in this study.

## Results

The following subsection headings are deliberately chosen to resemble a manual that could belong to any other digital device connected to a data network, such as a smartwatch. The cardiac monitor represents the sensor that is integrated into a data network, connecting it to the company's servers and to healthcare providers. By describing remote cardiac monitoring as a data network, I will illustrate the modus operandi of the data network, focusing on how different temporalities and data types external to the monitoring system must be synchronized to make the remotely collected data useful for diagnosis. Furthermore, I will show how the imaginary of remote cardiac monitoring as an automatically synchronized data network does not match with everyday data practices. The difference between the patients' perception of continuous care and the uncoupling of data collection, processing, and interpretation creates an illusion. Instead of the traditional medical appointment reuniting the patients' narratives, the cardiologists' expertise, and measured evidence, remote cardiac monitoring will disrupt the usual feedback loop between patients and doctors. Drawing on the analytical framework of temporalities, I conceptualize this as the illusion of immediacy.

### General instructions

Cardiologists in Switzerland may recommend remote cardiac monitoring to patients who are experiencing symptoms they suspect are related to a heartbeat that is too slow, too fast or irregular. Another important group are patients who have suffered a stroke of unknown cause. When conventional examinations fail to detect the suspected arrhythmia, long-term remote cardiac monitoring is the last possibility to eventually detect it. A cardiologist told me that it serves to identify a potentially “hazardous seed”, while the market-leading biomedical company promotes remote cardiac monitoring as the possibility to “unlock the answer”. Although it is possible to have a cardiac monitor inserted without being connected to remote monitoring, cardiologists and companies strongly encourage adherence. Algorithms become more accurate as they are trained on large amounts of data, so medical and commercial stakeholders are interested in accumulating data and, therefore, in patients who adhere to remote monitoring. Patients sign a general consent for data sharing. Doubts about privacy are very rare among them, and when they do, they express them in the form of jokes, for example by comparing it to a cat microchip. Overall, the hope of finally finding out what was wrong with them outweighed privacy concerns. Specifically, the telemedical follow-up is promoted by healthcare professionals to patients by pointing out the possibility of immediate feedback compared to a manual download at the hospital during a calendar-based follow-up that would take place every 3 or 6 months. Nurses and cardiologists explained to patients that the remote monitoring system would regularly transmit any relevant episode of irregular heartbeat. As a result, potential treatment could be implemented in a timely manner. Another argument made by nurses and cardiologists is the time saved by patients not having to come to the cardiology department every 3 or 6 months.

### Assemblage

The insertion of the cardiac monitor requires a minor surgical procedure and is usually performed at the bedside by cardiologists or specially trained nurses. Compared to other heart surgeries, it is a minimally invasive procedure because the device is placed just under the skin without a direct connection to the heart. The procedure itself takes only a few minutes, but preparation can take up to an hour. Because of the cardiologists' busy and unpredictable schedule, patients may have to wait a few hours before he or she is available to perform the insertion. Specially trained nurses who can perform the insertion can reduce the patients' waiting time. Before the insertion, the spot on the chest is sensed by touch, shaved, if necessary, marked with a drawn arrow, and disinfected. The patients are then covered with a sterile fenestrated drape. Patients receive local anesthesia. Nurses and cardiologists typically describe the anesthetic injection as similar to a dentist's to prepare the patients for the burning pain that local anesthesia initially causes. The local anesthetic takes longer to take effect than the actual insertion of the cardiac monitor. First, the nurses or cardiologists use a cutting tool to make a small incision of about 1 cm. The applicator is then carefully inserted under the skin to serve as a placeholder for the cardiac monitor, which is then placed under the skin by manipulating the applicator. Finally, the applicator is removed, and the wound is sutured or glued. Most patients are surprised at how little time it takes to insert the monitor.

### Setup

After insertion, the patients and the cardiac monitor are connected to the remote monitoring system. It was this moment that stuck with me from the beginning of the ethnographic observations, and which also gave me the idea for this article. The nurses or cardiologists placed a company-specific “device reader” on the dressed wound of the freshly inserted cardiac monitor. They then used a company-specific computer or tablet to connect the implant, the patients' personal information, and the remote monitoring system. They would usually comment on this action by saying that they were now “programming” it. Inevitably, the image of a cyborg came to mind. But instead of just looking at the patients, I decided to look at the system as a whole. From that perspective, this scene represented the moment when the patients and the cardiac monitor were connected to a data network. It was simply like adding another data-collecting sensor to a pre-existing data network, centralized by corporate servers and accessible by healthcare providers. This image reminded me of other data networks in our everyday lives, where devices are constantly being added to or removed from other devices or data networks, such as connecting a speaker to a friend's smartphone.

Patients then receive final instructions before leaving the hospital. They receive the transmitter, which is responsible for automatic data transfer. Again, the cardiac monitor must be manually connected to the transmitter. The nurses or cardiologists guide this process step-by-step. Once the devices are successfully connected, they explain remote monitoring in detail. They focus on the easy handling, the automatic data transfer and that the transmitter must be plugged in near the bed, ideally at the bedside table. Often nurses or cardiologists give them the simple advice: “Just plug it in and forget about it” (fieldnotes, both hospitals). This is intended to reassure patients, especially if they do not feel comfortable using a technological device correctly. The patients are then discharged with the cardiac monitor placed under the skin and the transmitter packed in a cardboard box. In general, and if the patients have no follow-up due to other comorbidities, remote reading works on the principle of "no news is good news. Nurses and cardiologists told patients that they would be contacted as soon as something was found (field notes, both hospitals). This means that patients will only hear from the hospital if the cardiac monitor detects an arrhythmia that the tele-nurses or cardiologists deems clinically relevant. One of the two hospitals I visited has adopted the practice of calling the patients the day after the insertion to inform them that everything is working as is it should.

### Operation mode

Before I present the perspectives of the nurses who handle the data and the patients, I will illustrate the automated processes of data collection and transmission by tracing the path from the moment the heart beats irregularly to the moment this irregular beat is taken into consideration at the hospital.

Let's imagine a patient, named Sandra. She had received a cardiac monitor 10 months ago following a stroke of unknown cause. The cardiologists suspect atrial fibrillation as the cause. After the stroke, she continued to work with a reduced workload. Today, she had lunch with her co-workers as usual. Now she is sitting comfortably at a table, having coffee with them before going back to work. The room is filled with laughter. She feels a little tired. Unnoticed by her, the upper and lower chambers of her heart are beating out of sync for a few minutes. But the algorithms in the cardiac monitor detect the irregular electrical signals by comparing them to the millions of heart rhythm sequences with which they have been trained. As a result, this sequence of atrial fibrillation is recorded and stored in the cardiac monitor. Sandra finishes her workday, goes home, and does not stay up too late because she still feels a little tired, not thinking of any potential harm. After midnight, the transmitter next to her bed automatically connects via radio frequency or Bluetooth to her inserted cardiac monitor. The recording is then transmitted to a company-provided online server via a landline telephone or wireless cellular network. The next day, tele-nurses log into a software program provided by the company and reviews the recordings. Recordings from cardiac monitors are considered the least important compared to other connected devices such as defibrillators or resynchronization therapy devices. Consequently, and depending on the total daily number of alerts sent, the recordings coming from cardiac monitors will be checked only in the afternoon. If considered relevant by the tele-nurses, the recording is forwarded and/or discussed with the attending cardiologist, who makes the final decision on whether or not to act on it.

### Data processing

Every time the algorithms detect an irregular heartbeat, or a series of signals interpreted as such, an alert is sent via the transmitter to the remote monitoring system. Yet, data do not speak for themselves. It is only in the specific context of a patient that it may or may not make sense. It is the task of specialized tele-nurses to correlate the recorded data with the patient's case. The telemedical follow-up takes place during office hours, Monday through Friday. In the morning, a tele-nurse enters the office and starts her computer. With her personal login she has access to the telemedical platforms provided by the different biomedical companies. The platforms are similar to an email inbox, displaying the latest alerts from the cardiac monitors and other cardiac devices. Some of them can be color-coded, like a traffic light, to indicate the level of importance. The thresholds for the different types of arrhythmias, which are set by default by the companies, can be reprogrammed by the healthcare professionals. The number of data recordings to be processed varies from day to day. Currently, they have to check about a hundred alerts daily including therapeutic and diagnostic devices (usually a bit more on Mondays as data accumulates over the weekend). Moreover, the high sensitivity of the cardiac monitor generates a significant number of false-positive alerts (Afzal et al., 2020). This is not the only time-consuming part of the data review process.

At first glance, the tele-nurses use detailed knowledge of the patients' health status and often also of the patients' everyday life and hobbies to contextualize a recorded heart rhythm episode. In this case, knowledge means recognizing the name and/or type of arrhythmia transmitted by a connected cardiac device. Over time, they acquire a detailed knowledge of the connected patients and the frequency with which their cardiac device sends an alert. As a result, they will learn which patients are prone to false-positive alerts and adjust the processing of these data accordingly.

One ethnographic observation provides an illustrative example. Once, when I was sitting in the telemedical unit, a tele-nurse showed me an episode of palpitations. The episode indicated 180 beats per minute. Recognizing this patient by its name, the tele-nurse told me that in this specific case, the arrhythmia episode was nothing serious. I asked her how she could be sure about that. She drew my attention to the time the episode was recorded and said:

“Look, here he was cycling again. At this time of day, he always uses his stationary bicycle. We know that. It's normal if you are doing sports” (tele-nurse, 53 years old).

Nevertheless, the algorithm of the cardiac monitor systematically marked this episode as potentially relevant, because of the fast heartbeat. The tele-nurse told me that this is typically an alert to be discarded. She went on in her explanation, telling me that this is the main challenge of her everyday work: distinguishing between relevant and irrelevant episodes, contextualizing them with the patients' background knowledge, and not missing any important indication.

If the tele-nurses are unsure about a recording, they will still click on it to see the recorded heart rhythm in detail. They will look closely at the graph representing the heartbeat to see if the algorithm has missed or overidentified a particular moment in the heart contraction. Sometimes, they will use a calculator to manually calculate the heartbeats per minute. If they need further clarification, they can ask their colleagues or the cardiologists in charge to examine the recorded episode more closely. The responsibility for correctly interpreting the recorded data makes their job exciting, but it also positions them as a critical node in the remote monitoring data network. One tele-nurse explained this ambivalence in the interview as follows:

“It's really never boring. Every day there is the suspense: What will I find today? Amongst us we say: What will I catch today? (...) But if you don't see it, it will be lost” (tele-nurse, 55 years old).

Even though patients are made aware that remote cardiac monitoring is for monitoring only, not for emergency intervention, tele-nurses play a central role in establishing a diagnosis. If they miss a decisive alert, there will be a delay in diagnosing a potentially life-threatening condition. Accordingly, one nurse referred to the task of reassembling, linking, and interpreting different types of data to understand the transmitted episode as “detective work.” The fine knowledge of how to examine recorded episodes or learning which cardiac monitors regularly recorded false positives was acquired over time. Consequently, they would directly discard an alert according to a name and/or a type of arrhythmia without further investigation if they recognized it as a repeated artifact.

Sometimes the tele-nurses would call a patient to verify the situation in which the episode was recorded. They would first ask if the patients were feeling well or if they had noticed anything unusual the day before the episode was recorded. Together with the patients and the time the episode was recorded, they reconstruct what they were doing at that moment the day before. Thereby, the automatically recorded data was connected to the patients' sensations and/or actions. This additional information helped the tele-nurses to either discard the alert or make a note for further discussion with the cardiologists.

However, the tele-nurses did not contact the patients unless it was necessary or asked for by the cardiologists. The sovereignty of data interpretation, knowledge production, and the decision to communicate it to the patients remained clearly in the hands of the tele-nurses and the cardiologists. They justify this approach by saying that they want to prevent patients from becoming anxious. In one hospital, a one-page written report is sent to patients every 3 months. However, the healthcare professionals refrain from communicating every arrhythmia recorded, as this tele-nurse explained:

“For example, we have many ventricular tachycardias, but they are self-limiting. Or we see many, frequent ventricular extrasystoles. We don't write the supraventricular stuff in the report. So, if it's not atrial fibrillation, it worries patients when you write that they had supraventricular tachycardia” (tele-nurse, 55 years old).

She told me, that she had once mentioned the recording of a “supraventricular tachycardia” in a report. The patient had immediately called the telemedical unit immediately after receiving the report and asked for clarification. It took a lot of time to explain to the patient that this episode was part of the non-dangerous arrhythmias. Hence, they keep these events confidential and communicate only if they judge it appropriate from a clinical point of view. However, not all tele-nurses do fully agree with this practice, but they have to follow the rules set by the cardiologists. This illustrates that the main source of frustration was not the data produced as such (Pols, 2012), but the different views on how to deal with them.

### User satisfaction

Overall, patients and healthcare professionals perceived the inserted cardiac monitor as reassuring. The fact that the patients' heart rhythm was continuously monitored, regularly transmitted, and reviewed by healthcare professionals by healthcare professionals was reassuring to all users. For all involved actors remote cardiac monitoring is a way of taking seriously the unexplained symptoms and the uncertainty associated with their possible recurrence (Nettleton et al., 2004).

This effect was particularly strong for potential arrhythmias that patients are unlikely to notice, such as atrial fibrillation, which is a risk for recurrent stroke. Patients and healthcare professionals alike were convinced that these irregularities would be detected by the cardiac monitor and could subsequently be taken into adequate consideration. I call this phenomenon the “reassuring effect” of remote monitoring. On the one hand, this effect resulted from the perception of constantly being cared for by a healthcare professional. On the other hand, it was related to a reduction in the feeling of uncertainty about unexplained symptoms. Although the cardiac monitor could not intervene to prevent another symptomatic episode of major (e.g., stroke) or minor (e.g., fainting) impact on their lives, patients appreciated the feeling of being in control, while cardiologists appreciated the feeling of having at least some control over the situation. One cardiologist described remote cardiac monitoring as a kind of digital ties reassuring her in a situation of diagnostic uncertainty:

“It is reassuring for the doctor to say, ‘I've set up everything I could. I keep these ties.' Personally, I consider them as ties, like protections for the patients. To reassure the patients but also to reassure yourself, so that we do not lose the patients in the “wilderness.” So, the patients are still being monitored. It is a kind of double psychological effect, but especially for the doctor” (cardiologist, 43 years old).

Her description of digital ties fits well with the imaginary of a data network. However, tele-nurses and cardiologists had a privileged access to the data compared to the patients who could not see whether their cardiac monitor had detected and transmitted an heart rhythm recording or not. Consequently, they depended on the feedback from the healthcare professionals to know about potential data transmissions. Nevertheless, a reassuring effect was established just by the imaginary of being permanently connected to the hospital. Adding up to the previous findings of Pols (2012), the reassuring effect persisted even if there was not much contact between patients and tele-nurses or cardiologists. A patient who has had a cardiac monitor for two and a half years after having two unexplained ischemic attacks said about the implant:

“I would like to say that I have been really happy about this thing. This gave me some kind of certainty for at least two and a half years. That was actually true. Being monitored made me feel safe. There is someone in the hospital who is looking after me. Even if there wasn't much direct contact” (woman, 66 years old).

For patients like this woman, the digital connection was enough to provide a sense of reassurance. Interestingly, this effect was sometimes even more present among patients' family members who were worried about their loved ones, as this example shows:

“My sons and my husband said: Be happy, if there is something, they will immediately sound the alarm. Even if you would not notice it” (woman, 82 years old).

The reassuring effect of being cared for was particularly strong in the first few months after insertion. However, the persistence of this reassurance depended on the system working as promised. Patients expected to receive a call from the hospital within a short time if their cardiac monitor recorded an event, as they were told by nurses and cardiologists during the instruction after the insertion. Several patients expressed disappointment when a recorded episode was not handled as expected. In the following quote, a patient told me how he complained when he was not informed immediately, and how the second time it went as expected:

“The first episode was recorded on October 11, but I did not receive the [written] report until October 21, when I was asked to see my cardiologist. I had already called my cardiologist to report the incident. Then she apologized. The next time, my cardiologist called me directly about an episode that had happened the day before. That was the confirmation for me, ‘Okay, it can work right away if needed.' The first episode probably got stuck somehow. (...) It was probably a unique situation. I work in healthcare myself and I know how it works with accounts and reports. It falls on the staff, who then have to deal with all of that” (man, 65 years old).

His experience illustrates the expectations patients have toward remote cardiac monitoring. Moreover, it shows that a bad experience can be compensated by a later one which meets this patient's expectations. Contrary to what he believed, receiving “delayed” feedback or no feedback at all was not his unique experience. It was a recurrent topic among the interviewed patients.

However, to be disappointed or reassured by remote cardiac monitoring, patients needed to know that an event had been transmitted. Typically, heart rhythms are monitored using trained and self-learning algorithms that automatically record abnormal heart rhythms. The bedside transmitter shows only an “ok” sign and the date of the last successful transmission, which usually happens automatically once a day. Because no other information is provided, some patients were concerned in the first weeks or months after insertion whether the telemonitoring system was really working. Some of them regularly checked the screen to see if the data transmission had occurred. If the displayed date had not been updated, they performed a manual data transmission using the “device reader”, as shown in the example of this patient:

“I think recently I had to do it manually three times in a row. It did not do it at night. I don't know why. I don't sleep so well anymore. Or haven't slept so well now with the last chemotherapy. When I wake up at night, I press the button to check it briefly, and then it connects. Then it does [the transmission]. The last three times, I had to hold [the reader] onto [the insertion spot]. [The transmission] is kind of set somehow between 12 and 5 in the morning. I woke up too late and did it manually. But that's not a problem” (man, 60 years old).

This patient, like some of the other interviewees, wanted to make sure that the recordings were transmitted, especially during the first few months after insertion. This woman described in the interview how her behavior changed after a first arrhythmia was diagnosed and treated with ablation:

“Somehow, I had this feeling in the beginning: It's mega important that I check if it's really been sent and all that. Afterwards, after the first ablation, I felt like: Well, well, it's all right. (...) I checked maybe every three months to see if it had really been sent. And most of the time it had been sent. However, in the first year, I had checked it almost every day or at least every second or third day” (woman, 23 years old).

Like other study participants, she initially wanted to make sure that data was being transmitted regularly. This does not correspond to the “just plug it in and forget about it” advice given to patients by nurses or cardiologists during instructions after the insertion. These patients' experiences also illustrate the importance of the materiality of the transmitter, which, unlike the cardiac monitor, is a visible device in their bedroom that allows them to check (at least in a very simple way) whether the system is working or not (Weiner and Will, 2018).

Additionally, some patients were given a third device looking like a remote control with which they could actively force their implant to record an episode. The idea behind this device is to generate a link between an embodied experience and a potential heart arrhythmia. This allows patients to mark a heart rhythm episode in a moment in which he or she experiences symptoms of unease for example. However, the possibility of deliberately forcing and transmitting a recording during a moment of discomfort raised high hopes for receiving feedback. The experience of this patient who regularly felt disturbed in her everyday life by the sensation of extrasystoles shows her disappointment about the lack of feedback:

“So, I would set off alerts precisely because of [the extrasystoles]. And then, well, what annoyed me was that I never got any feedback. In fact, yes, later [the cardiologist] reassured me by telling me that it wasn't serious. Still, it would have been reassuring for me to be contacted when I launch an alert. Not within an hour, because it's true, it has happened quite often over the weekend. After I had been in contact with the other doctor, I often set it off to show how frequently it happened. He had told me to do so every time I feel something. So, I did it, but then I didn't get any answer to that. I would have liked someone to call and tell me that there was nothing, nothing to report, nothing serious, you know. Just to reassure me. So, afterwards, I asked myself: well, what's the point of having this, if, when setting off an alert, I have no news, no follow-up. So, then they said, in fact if there's no problem, we won't call you” (woman, 58 years old).

To avoid frightening patients and to be cost effective, patients are contacted only when the nurses or cardiologists choose to do so. Thus, there is no follow-up for manually recorded episodes that show no irregularities. However, my qualitative interview data underscores the importance of feedback for monitored patients. They often felt cut off from the feedback loop.

Moreover, the reassuring effect generally diminished over time. Most patients were less reassured by the remote cardiac monitoring system when I met them for the second interview. This is well illustrated by the example of this patient, whom I asked during the second interview if the reassuring feeling was still as present as during our first interview:

“Yes, that is a good question. I was thinking about that today. I thought you would ask me that (both laugh). It is true I have said in the beginning, “Now, I am totally monitored.” It also gives me a good feeling. Yes, it is still a bit there in the sense of which I have talked about before. If I had [another stroke] now, maybe I'd see it differently. Or maybe there is something [like an arrhythmia] that I do not know about. In fact, that is probably what is preventing me from having [the cardiac monitor] taken out” (woman, 68 years old).

This quote, as well as the one above, shows how important it is for patients to continue to feel well cared for. When a cardiac monitor is placed, there is great hope that the cause of unexplained symptoms will be found. If the cardiac monitor fails to detect arrhythmias for several months, these hopes are dashed. Similarly, patients who did not receive feedback on episodes they deliberately marked to show the cardiologists when they felt unwell began to question the usefulness of remote cardiac monitoring.

## Discussion

In this article, I have illustrated how data-intensive medicine changes the way a diagnosis is made. Thinking of medical practice as a data network illustrates how the spatial and temporal uncoupling of processes that used to happen in one place creates an “illusion of immediacy”. Instead of being automatically in sync as one would expect it from other data networks, the simultaneity or closeness (Pols, 2012) of the elements constituting a diagnosis need to be put into sync by a human. This article explores the coexistence of multiple temporal dimensions in data-intensive medicine by examining the experiences of patients, tele-nurses, and cardiologists with remote cardiac monitoring in Switzerland. In the following paragraphs, I will discuss the four main findings based on ethnographic observations during the insertion procedure and in the telemedical unit of two university hospital, as well as longitudinal and retrospective interview data.

First, the tele-nurses have a central position within the data network of remote cardiac monitoring. Her role is crucial, because data do not speak for themselves, but must be interpreted in relation to the patient's lifeworld (Grew and Svendsen, 2017). Although the data recorded by the monitor is automatically transmitted and synchronized through the telemedical system provided by the company, they need to be further synchronized with other data types, such as the last hospital visits, co-morbidities, and/or the actual situation in which the recording has been produced. Hence, this article suggests that the use of data-intensive technologies for diagnosis increases the need for human synchronization (Elias, 1992). Contrary to the traditional follow-up during which simultaneity is given by a shared space, the network-like character of data-intensive medicine can only produce meaningful knowledge if the links between the different types of data are correctly put into sync by a human (Weinberger, 2011). This requires “detective work” as the interviewed tele-nurses called it. But it did not just involve consulting the right documents to gather the relevant information. Over time, the tele-nurses acquired a fine knowledge about the patients' habits which helped them to faster process false-positive alerts (Piras and Miele, 2019). For example, they knew that a certain patient always uses his stationary bicycle at a certain time of the day which resulted in an alert of an abnormally high pulse. Consequently, they always dismissed this alert without further examination. This suggests that a certain form of intimate knowledge is indispensable for medical decision-making. However, the way remote cardiac monitoring is uncoupling the processes of data collection, analysis, and interpretation shifts the balance of power in favor of medical professionals, thus calling into question the promise of the participatory dimension of “personalized medicine.”

Second, the setup of data-intensive technologies such as the cardiac monitor resembles that of any other connected devices, for example smartwatches. Consequently, receiving a cardiac monitor conveys the imaginary of being in sync with the hospital and therefore the clinician. Even though most patients know that remote monitoring does not work like an emergency system, they were reassured by the idea of being constantly connected to the clinic. This article shows how remote cardiac monitoring has a subjective reassuring effect on both patients and healthcare professionals. While patients felt that they were being “continuously cared for,” healthcare professionals perceived it as “caring well” for their patients. This reassuring effect resulted from the imaginary of the digital ties provided by remote cardiac monitoring. For patients, however, it was precisely this reassuring effect that disappeared over time or was even a source of disappointment when the technology did not live up to the imaginary of a synchronized data network (Petersen, 2015).

Third, this gap between the reassuring effect based on the imaginary of a data network in snyc allowing for prompt feedback after an alert of an arrhythmia, and the above-described human synchronization work, which takes time, led to what I call an “illusion of immediacy”. Although the inserted sensor monitors the heart round the clock, data transmission only happens once a day, and the tele-nurses work only during office hours from Monday to Friday. Consequently, if the heartbeat stops for a few seconds on a Saturday morning, the recorded episode will not be seen until Monday morning at the earliest. Some patients have expressed disappointment in not receiving immediate feedback or no feedback at all. Similar to the introduction of the telephone into the doctor's office, data-intensive medicine conveys the notion of a doctor-patient connection that is available 24/7 (Greene, 2022). Although technically feasible via the data network, healthcare professionals need time to accurately link and interpret the recorded data to produce meaningful knowledge. This reconfiguration of the temporal dimension of diagnostic work through patient remote monitoring may also affect the role and value of the gut feeling in everyday clinical practice (Kristensen et al., 2021).

Fourth, data-intensive technologies like the cardiac monitor uncouple the traditional diagnostic procedure of anamnesis, examination, and discussion of the results between the cardiologists and the patients. My article shows that the setup of remote cardiac monitoring fosters healthcare professionals to hold back on patient feedback. As it is precisely the role of the doctors or cardiologists to come up with a conclusive diagnosis (Groopman, 2008), they do not communicate every arrhythmia episode with the patients to prevent them from becoming anxious. Moreover, they neither comment on episodes recorded and sent deliberately by patients while having symptoms if they do not show any abnormal rhythms according to clinical standards. This illustrates how the spatial and temporal uncoupling of data collection, its processing and interpretation in remote cardiac monitoring leads to a privileged data access for healthcare professionals making them the main users of the data network (Oudshoorn and Pinch, 2003). However, this may lead to disappointment among patients in the long run, especially if they make efforts to control and ensure data transmission, thereby becoming “diagnostic agents” (Oudshoorn, 2011). The sovereignty of data interpretation, knowledge production, and the decision to communicate with the patients remains within the walls of the clinic, thus devaluing patient work and participation (Oudshoorn, 2008). This is in line with the vision of “personalized medicine” focusing on data first, and on patients second (Vogt et al., 2016; Prainsack, 2017; Hoeyer, 2019). Future studies should aim to carefully disentangle how different types of big data sets are combined and who has the power to collect, process, and interpret them (Canali and Leonelli, 2022).

Looking at data-intensive medicine from the angle of a data network (Weinberger, 2011), instead of a classic care infrastructure (Weiner and Will, 2018), was useful to disentangle the multiple temporal dimensions co-existing in patient remote monitoring. In general, the temporal aspects of data-intensive medicine are not yet well-understood. While the patients and their embodied experience are always in sync, maybe except for sleep, this is not the case for the clinical examination. The promise of data-intensive technologies like the cardiac monitor is to put the patients and the clinic in one data network. But simply connecting the two is not enough. There is a need to put different types of data and different time frames into sync to render the endeavor meaningful for patients and doctors.

### Limitations

The patient profiles of the participants were very different in terms of their clinical history (congenital heart disease, unexplained arrhythmias, stroke, comorbidities, etc.), which influenced the importance they attached to the cardiac monitor in their lives. However, it was the observation during this fieldwork that all these clinically very different patients share similar concerns, especially the lack of regular feedback. Although I tried several times to recruit a patient who had refused the insertion of a cardiac monitor, I was not successful. According to the cardiologists interviewed, very few patients completely refuse remote cardiac monitoring.

## Conclusion

Data-intensive medicine privileging easily quantifiable information over unstructured patient narratives promises to improve healthcare through bigger and more comprehensive data sets. However, the production of these types of data sets uncouples the processes of data collection, analysis, and interpretation which previously took place within a single medical site. However, to make these data sets meaningful and useful, human synchronization of the multiple data types and time frames involved is required. This article on remote cardiac monitoring in Switzerland illustrates how the diagnostic process changes when the data is no longer collected in discrete moments in the cabinet or the clinic but continuously and remotely. The network-like character of patient remote monitoring conveys the perception of continuously “being cared for” among patients, and constantly “caring for patients” among healthcare professionals. On the one hand, this results in a reassuring effect among for patients and healthcare professionals alike. On the other hand, patients lose this reassurance over time or especially if their expectation of a prompt feedback is not met. I call this phenomenon the “illusion of immediacy”. Although medical practice increasingly relies on data, the data only makes sense if it is properly linked to other information, such as the situation in which it was recorded. Tele-nurses play a central role in doing the “detective work” to make the data meaningful to the cardiologists and, ultimately, to the patients. The knowledge generated by these networked data is the decisive element for data-intensive medicine to generate a diagnosis which might not be made as immediately as promised, but—with a bit of a chance—sooner than with conventional discrete measurements.

## Data availability statement

The anonymized data supporting the conclusions of this article will be made available by the authors, without undue reservation.

## Ethics statement

The studies involving human participants were reviewed and approved by Commission cantonale d'éthique de la recherche sur l'être humain (Cantonal commission on ethics in human research of the canton Vaud). The patients and healthcare professionals provided their written informed consent to participate in this study.

## Author contributions

The author confirms being the sole contributor of this work and has approved it for publication.

## References

[B1] Afzal M. R. Mease J. Koppert T. Okabe T. Tyler J. Houmsse M.. (2020). Incidence of false-positive transmissions during remote rhythm monitoring with implantable loop recorders. Heart Rhythm 17, 75–80. 10.1016/j.hrthm.2019.07.015 31323348

[B2] Armstrong D. (1995). The rise of surveillance medicine. Sociol. Health Illness 17, 393–404. 10.1111/1467-9566.ep10933329

[B3] Braun V. Clarke V. (2021). Thematic Analysis: A Practical Guide. London: SAGE.

[B4] Canali S. Leonelli S. (2022). Reframing the environment in data-intensive health sciences. Stud. History Philos. Sci. 93, 203–214. 10.1016/j.shpsa.2022.04.006 35576883

[B5] Cesario A. Lohmeyer F. M. D'Oria M. Manto A. Scambia G. (2021). The personalized medicine discourse: archaeology and genealogy. Med. Health Care Philos. 24, 247–253. 10.1007/s11019-020-09997-6 33389365

[B6] Deftereos S. Papoutsidakis N. Giannopoulos G. Kossyvakis C. Lekakis J. (2016). Remote monitoring of the cardiac rhythm: where do we stand today? Continuing Cardiol. Educ. 2, 168–175. 10.1002/cce2.36

[B7] Elias N. (1992). Time: An Essay. Oxford: Basil Blackwell.

[B8] Erikainen S. Chan S. (2019). Contested futures: envisioning “personalized,” “stratified,” and “precision” medicine. N. Genet. Soc. 38, 308–330. 10.1080/14636778.2019.1637720 31708685 PMC6817325

[B9] Greene J. A. (2022). The Doctor Who Wasn't There: Technology, History, and the Limits of Telehealth. Chicago, IL: University of Chicago Press.

[B10] Grew J. C. Svendsen M. N. (2017). Wireless heart patients and the quantified self. Body Soc. 23, 64–90. 10.1177/1357034X16663005

[B11] Groopman J. (2008). How Doctors Think. New York, NY: Mariner Books.

[B12] Hoeyer K. (2019). Data as promise: reconfiguring danish public health through personalized medicine. Soc. Stud. Sci. 49, 531–555. 10.1177/0306312719858697 31272287

[B13] Jones D. S. (2013). Broken Hearts: The Tangled History of Cardiac Care. Baltimore, MD: JHU Press.

[B14] Kalahasty G. Alimohammad R. Mahajan R. Morjaria S. Ellenbogen K. A. (2013). A brief history of remote cardiac monitoring. Cardiac Electrophysiol. Clin. 5, 275–282. 10.1016/j.ccep.2013.06.002

[B15] Kristensen B. M. Andersen R. S. Nicholson B. D. Ziebland S. Smith C. F. (2021). Cultivating doctors' gut feeling: experience, temporality and politics of gut feelings in family medicine. Cult. Med. Psychiatry 46, 564–581. 10.1007/s11013-021-09736-3 34564779

[B16] Mackintosh N. Armstrong N. (2020). Understanding and managing uncertainty in health care: revisiting and advancing sociological contributions. Sociol. Health Illness 42, 1–20. 10.1111/1467-9566.13160 32757281

[B17] Maillard N. Perrotton F. Delage E. Gourraud J.-B. Lande G. Solnon A.. (2014). Cardiac remote monitoring in France. Arch. Cardiovasc. Dis. 107, 253–60. 10.1016/j.acvd.2014.02.004 24709285

[B18] Moers W. (2008). The City of Dreaming Books. New York, NY: Abrams.

[B19] Nettleton S. O'Malley L. Watt I. Duffey P. (2004). Enigmatic illness: narratives of patients who live with medically unexplained symptoms. Soc. Theory Health 2, 47–66. 10.1057/palgrave.sth.8700013

[B20] Oudshoorn N. (2008). Diagnosis at a distance: the invisible work of patients and healthcare professionals in cardiac telemonitoring technology. Sociol. Health Illness 30, 272–288. 10.1111/j.1467-9566.2007.01032.x 18290936

[B21] Oudshoorn N. (2011). Telecare Technologies and the Transformation of Healthcare. Basingstoke: Palgrave Macmillan.

[B22] Oudshoorn N. Pinch T. (eds.) (2003). “Introduction: how users and non-users matter,” in How Users Matter. The Co-construction of Users and Technology (Cambridge: MIT Press), 1–25.

[B23] Petersen A. (2015). Hope in Health: The Socio-Politics of Optimism. Basingstoke: Palgrave Macmillan.

[B24] Petersen A. (2018). Digital Health and Technological Promise: A Sociological Inquiry. Abingdon: Routledge.

[B25] Piras E. M. Miele F. (2019). On digital intimacy: redefining provider–patient relationships in remote monitoring. Sociol. Health Illness 41, 116–131. 10.1111/1467-9566.12947 31599992

[B26] Pols J. (2012). Care at a Distance: On the Closeness of Technology. Amsterdam: Amsterdam University Press.

[B27] Porter T. M. (1995). Trust in Numbers: The Pursuit of Objectivity in Science and Public Life. Princeton, NJ: Princeton University Press. 10.1177/030631299029004007 11623934

[B28] Prainsack B. (2017). Personalized Medicine: Empowered Patients in the 21st Century? New York, NY: NYU Press.

[B29] Rosenberg C. E. (2002). The tyranny of diagnosis: specific entities and individual experience. Milbank Q. 80, 237–60. 10.1111/1468-0009.t01-1-00003 12101872 PMC2690110

[B30] Ruckenstein M. S. Schull N. D. (2017). The datafication of health. Annu. Rev. Anthropol. 46, 261–278. 10.1146/annurev-anthro-102116-041244

[B31] Schweizerische Herzstiftung (2022). Herzrhythmusstörungen. Schweizerische Herzstiftung. Available online at: https://swissheart.ch/erkrankungen-und-notfall/herzkrankheiten-und-hirnschlag/herzrhythmusstoerungen (Retrieved December 10, 2022).

[B32] Sysling F. (2020). Measurement, self-tracking and the history of science: an introduction. History Sci. 58, 103–116. 10.1177/0073275319865830 31726878 PMC7328690

[B33] Theodore P. M. (1995). Trust in Numbers: The Pursuit of Objectivity in Science and Public Life. Princeton, NJ: Princeton University Press. 10.1177/030631299029004007 11623934

[B34] Vogt H. Hofmann B. Getz L. (2016). The new holism: P4 systems medicine and the medicalization of health and life itself. Med. Health Care Philos. 19, 307–323. 10.1007/s11019-016-9683-8 26821201 PMC4880637

[B35] Weinberger D. (2011). Too Big to Know: Rethinking Knowledge Now That the Facts Aren't the Facts, Experts Are Everywhere, and the Smartest Person in the Room Is the Room. New York, NY: Basic Books.

[B36] Weiner K. Will C. (2018). Thinking with care infrastructures: people, devices and the home in home blood pressure monitoring. Sociol. Health Illness 40, 270–282. 10.1111/1467-9566.12590 29464773

